# Dexamethasone Inhibits Heparan Sulfate Biosynthetic System and Decreases Heparan Sulfate Content in Orthotopic Glioblastoma Tumors in Mice

**DOI:** 10.3390/ijms241210243

**Published:** 2023-06-16

**Authors:** Dmitry K. Sokolov, Oleg B. Shevelev, Anna S. Khotskina, Alexandra Y. Tsidulko, Anastasia V. Strokotova, Galina M. Kazanskaya, Alexander M. Volkov, Evgenii E. Kliver, Svetlana V. Aidagulova, Evgenii L. Zavjalov, Elvira V. Grigorieva

**Affiliations:** 1 Institute of Molecular Biology and Biophysics, Federal Research Center of Fundamental and Translational Medicine, Novosibirsk 630117, Russia; dmit_s95@mail.ru (D.K.S.); sephellone@gmail.com (A.Y.T.); anastasia-suhovskih@mail.ru (A.V.S.); g_kazanskaya@meshalkin.ru (G.M.K.); s.aydagulova@gmail.com (S.V.A.); 2 Institute of Cytology and Genetics, Novosibirsk 630090, Russia; shevelev.oleg.nsk@gmail.com (O.B.S.); dotcenko@bionet.nsc.ru (A.S.K.); zavjalov@bionet.nsc.ru (E.L.Z.); 3 E.N. Meshalkin National Medical Research Center, Novosibirsk 630055, Russia; a_volkov@meshalkin.ru (A.M.V.); kliver_68@mail.ru (E.E.K.); 4 Laboratory of Cell Biology, Novosibirsk State Medical University, Novosibirsk 630091, Russia

**Keywords:** glioblastoma, dexamethasone, temozolomide, glycosaminoglycan, heparan sulfate, heparan sulfate biosynthesis, glucocorticoid receptor, expression

## Abstract

Glioblastoma (GB) is an aggressive cancer with a high probability of recurrence, despite active chemoradiotherapy with temozolomide (TMZ) and dexamethasone (DXM). These systemic drugs affect the glycosylated components of brain tissue involved in GB development; however, their effects on heparan sulfate (HS) remain unknown. Here, we used an animal model of GB relapse in which SCID mice first received TMZ and/or DXM (simulating postoperative treatment) with a subsequent inoculation of U87 human GB cells. Control, peritumor and U87 xenograft tissues were investigated for HS content, HS biosynthetic system and glucocorticoid receptor (GR, *Nr3c1*). In normal and peritumor brain tissues, TMZ/DXM administration decreased HS content (5–6-fold) but did not affect HS biosynthetic system or GR expression. However, the xenograft GB tumors grown in the pre-treated animals demonstrated a number of molecular changes, despite the fact that they were not directly exposed to TMZ/DXM. The tumors from DXM pre-treated animals possessed decreased HS content (1.5–2-fold), the inhibition of HS biosynthetic system mainly due to the -3–3.5-fold down-regulation of N-deacetylase/N-sulfotransferases (*Ndst1* and *Ndst2*) and sulfatase 2 (*Sulf2*) expression and a tendency toward a decreased expression of the GRalpha but not the GRbeta isoform. The GRalpha expression levels in tumors from DXM or TMZ pre-treated mice were positively correlated with the expression of a number of HS biosynthesis-involved genes (*Ext1/2*, *Ndst1/2*, *Glce*, *Hs2st1*, *Hs6st1/2*), unlike tumors that have grown in intact SCID mice. The obtained data show that DXM affects HS content in mouse brain tissues, and GB xenografts grown in DXM pre-treated animals demonstrate attenuated HS biosynthesis and decreased HS content.

## 1. Introduction

Glioblastoma (GB) is the most aggressive human brain tumor, characterized by a high degree of malignancy and a poor prognosis [1]. Standard GB treatment consists of surgical resection, combined with adjuvant radiotherapy and chemotherapy with TMZ [2,3]. During the radiochemotherapy, DXM is used to prevent a treatment-induced brain edema, but its long-term administration causes serious side-effects such as Cushing’s syndrome, hyperglycemia and psychiatric symptoms, being a significant risk factor for poor overall survival of the GB patients [4,5,6,7]. As systemic drugs, TMZ and DXM affect both GB tumor and surrounding normal brain tissue, and an understanding of their molecular targets in brain might reveal new molecular mechanisms of their negative side-effects.

Among the numerous molecular targets for TMZ and DXM, they affect brain extracellular matrix (ECM) which is an important part of the GB microenvironment and has a significant impact on GB development and progression. To date, there is no doubt that the ECM should not no longer be regarded as a passive scaffold statically contributing to mechanical support in normal and pathological brain tissue, but as an active player in tumor-related activity [8]. The importance of the ECM role in normal brain tissue and gliomagenesis has resulted in much effort to implement ECM as a target and an instrument in the treatment of brain cancers [9,10].

A characteristic feature of the brain ECM is that it consists mainly of glycosylated macromolecules, such as proteoglycans (PGs), which play a critical role in brain physiology and cancer development [11,12,13]. The polysaccharide chains of PGs called glycosaminoglycans (GAGs) make a significant contribution to the functional activity of PGs and alterations of their composition/structure are often associated with various pathological conditions [14,15,16]. The important role of GAG in the development and pathological conditions of the nervous system is presented in the reviews [17,18,19]. In GB, significant changes in PG core proteins, GAG chains and their biosynthetic machinery occur, deciphering their tight involvement in GB development [20].

The data on the effect of TMZ and DXM on PGs and GAGs in different cells and tissues reviewed in [21] demonstrate an evident gap of knowledge on such effects in the brain. It has been shown that glucocorticoids affect the expression of PG core proteins in different experimental models—down-regulate neurocan and phosphacan expression in rat brains and primary cultured astrocytes [22]; affect expression of syndecan-1, glypican-1, brevican, versican, CSPG4/NG2, decorin, biglycan, lumican in rat brains in a dose- and a brain zone-dependent manner [23]; decrease agrin expression in mouse brains [24]; and increase the expression of syndecan-1, glypican-1, biglycan, and versican in SCID mouse brains in brain zone-dependent manner [25].

The effects of TMZ/DXM on the carbohydrate molecules of GAG are very poorly studied, and the published results relate mainly to their effect on chondroitin sulfate (CS). The local delivery of DXM in a nitrocellulose-based coating to rat brains reduces CS content one week post-implantation [26]; DXM decreases CS content by 2–2.5-fold in the rat brain cortex and increased CS content by 2-fold in the hippocampus [23]; DXM decreases CS content (−2.1-fold) in the subcortex of SCID mouse brain [25]. The DXM-induced changes in CS content in normal brain tissue are functionally significant, as demonstrated in the GB relapse animal model where the ex vivo DXM treatment of rat brain organotypic slices resulted in the accelerated adhesion of U87 GB cells to the slices, and the pre-treatment of SCID mice with TMZ and/or DXM resulted in the decrease in CS content in brain tissue and the more active growth of xenograft U87 GB tumors [25].

However, TMZ/DXM effects on heparan sulfate (HS) content in normal brain tissue and brain tumors remain even less studied, despite the important functional role of HS in brain physiology and pathology [27,28,29]. It was shown that HS expressed on tumor cells and in the tumor microenvironment regulates ligand-mediated signaling and promoting tumor cell proliferation and invasion, and these factors contribute to decreased tumor cell response to targeted RTK inhibition [30]; HS reduction in the vascular endothelium of the brain suppresses GB growth and neovascularization in mice [31]; and the increase in HS content in GB tissues represents negative prognostic factor for GB progression [32].

In our previous study, we presented the first data on the ability of DXM and/or TMZ to decrease HS content in both in the rat brain cortex and hippocampus [23]. The aim of this study is to investigate the effects of TMZ and DXM on HS content and its biosynthetic system in normal mouse brain tissue and GB xenograft tumors in a model of GB relapse in vivo.

## 2. Results

Being systemic drugs, TMZ/DXM possess not only a targeted effect on GB cancer cells but also negative side-effects towards normal brain tissue surrounding the GB tumor. To investigate such side-effects of TMZ/DXM in relation to peritumor brain tissue and their potential contribution to GB relapse, we developed an animal model that would approximately reproduce GB relapse development in real clinical practice. The routine treatment regimen for GB consists of the surgical removal of the tumor followed by radiochemotherapy using TMZ/DXM, and for to GB relapse occur, the GB cells remaining after surgery must survive in the brain tissue, which is constantly affected by these drugs. To study the contribution of the tumor microenvironment in this process, in our experimental model, normal mouse brain tissue was first exposed to TMZ/DXM followed by the inoculation of U87 GB cells in order to investigate what would happen to these GB cells in an intact or pre-treated brain microenvironment.

### 2.1. Dexamethasone Affects HS Content in Normal but Not Peritumor Mouse Brain Tissue 

**Figure 1:**
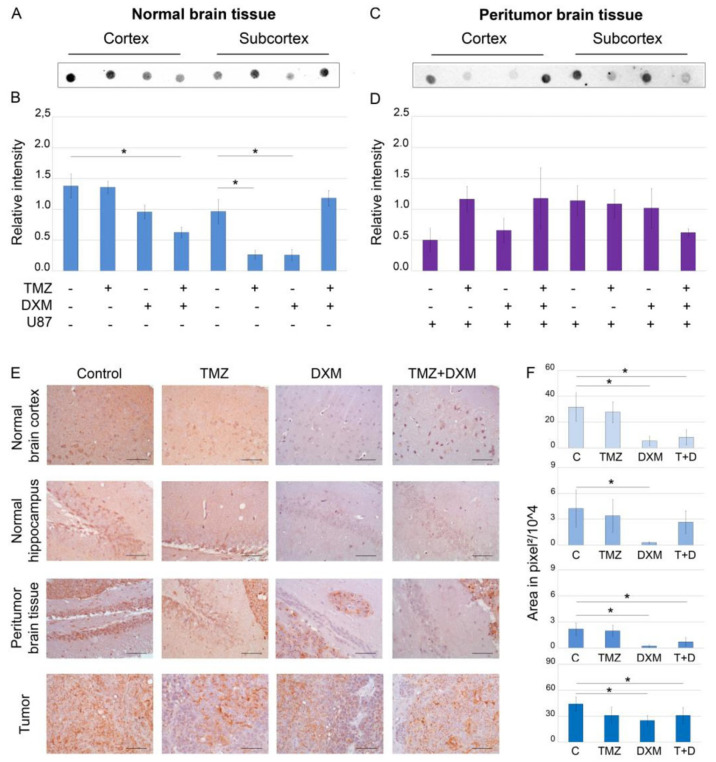
Heparan sulfate content in SCID mice brain tissues before and after treatments with TMZ and/or DXM. Dot blot analysis of the total HS content using anti-HS antibody in cortex (**A**,**B**) and subcortex (**C**,**D**) structures. (**A**,**C**) Original representative images. (**B**,**D**) Semi-quantitative analysis of the dot blots (ImageJ 1.52 software). (**E**) Immunohistochemical analysis of HS content in normal, peritumor and U87 xenograft tumor tissues. Magnification 400. Scale bars 50 µm. (**F**) Semi-quantitative analysis of HS content. The total area on the whole image of the positive IHC reaction in pixels^2^, a 500 × 500 area in pixels of the image related to the hippocampus is considered. Bars represent the mean ± SD from triplicate experiments (OriginPro 8.5). ANOVA + Fisher’s LSD test, *—*p* < 0.05. Control—non-treated mouse brain tissue; TMZ—temozolomide; DXM—dexamethasone.

To investigate the effects of TMZ and/or DXM on such a glycosylated component of normal and peritumor brain ECMs as HS, its content was determined by dot blot and immunohistochemical analyses using anti-HS antibodies (Figure 1). 

Dot blot analysis showed that DXM administration significantly decreased HS content (2–6-fold, *p* < 0.05) in all studied brain tissues—normal (Figure 1A,B,E,F), peritumor (Figure 1C–F) and U87 xenografts (Figure 1E,F). The observed effects were realized in a brain zone-specific manner—TMZ-DXM combination significantly decreased HS content in cerebral cortex, whereas TMZ or DXM did that for the subcortex structures significantly (-3-fold) in mono-regimens but not in combination (Figure 1A,B). Taking into account that TMZ had almost no effect on the HS content, the changes observed upon the administration of TMZ-DXM combination were apparently due to the DXM effect (Figure 1E,F). IHC data supported these results and demonstrated the capability of DXM to decrease HS content in the normal, peritumor and U87 tumor tissues (Figure 1E,F). As for the grown U87 xenografts, the inoculation of U87 cells into mouse brains pre-treated with DXM (or its combination with TMZ) resulted in a 1.5–2-fold decrease in HS content in the tumors (Figure 1E,F).

In summary, DXM shows an ability to reduce the HS content in the brain tissue of experimental SCID mice.

### 2.2. Pre-Treatment of Mice with Dexamethasone Inhibits the Transcriptional Activity of HS-Biosynthetic Enzymes in U87 Xenograft Tumors but Not Normal or Peritumor Brain Tissues

**Figure 2:**
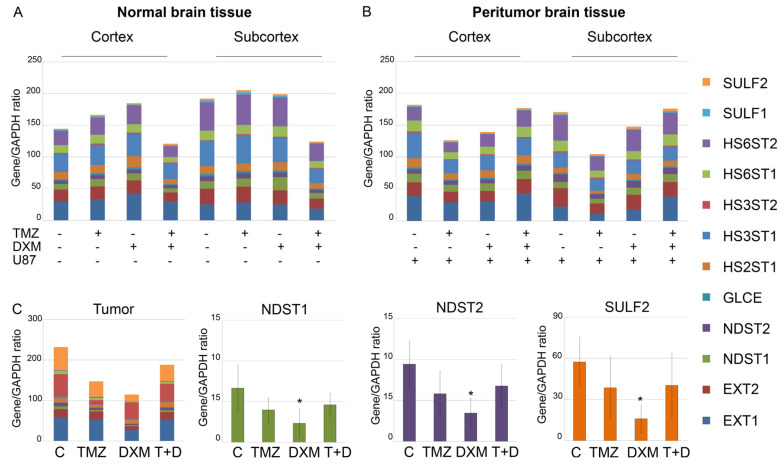
Transcriptional activity of HS-biosynthetic system in normal, peritumor and tumor tissues. mRNA levels of the studied genes (right panel) in normal (**A**) and peritumor (**B**) mouse brain tissues and U87 xenograft tumors (**C**) grown in the TMZ/DXM pre-treated animals. RT–PCR analysis, intensity of the amplified DNA fragments for each gene normalized to that of GAPDH. Stacked columns compare the contribution of each value to a total across categories. ANOVA + Fisher’s LSD test, *—*p* < 0.05. Controls—mouse brain tissue from untreated animals and those inoculated with U87 cells (for peritumor tissue); TMZ—temozolomide; DXM—dexamethasone.

Since HS biosynthesis occurs in the non-template manner and completely depends on the presence and activity of HS biosynthetic enzymes, the profiling of the transcriptional activity of HS biosynthesis-involved genes was performed (Figure 2). 

The overall transcriptional activity of the HS biosynthetic system was not significantly affected by either the TMZ/DXM treatment of the experimental mice (normal tissue) (Figure 2A) or the orthotopic inoculation of U87 cells alone or in combination with TMZ/DXM pre-treatment (peritumor tissue) (Figure 2B). However, the pre-treatment of the animals with DXM somehow affected the overall transcriptional activity of HS biosynthesis-involved genes in the grown U87 xenografts, revealing an ability of the mouse brain tissue to influence the molecular parameters of the inoculated U87 cells (Figure 2C). The demonstrated inhibition of HS biosynthetic system was mainly due to the selective significant suppression of the N-deacetylase/N-sulfotransferases *Ndst1* and *Ndst2* and sulfatase *Sulf2* (-3–3.5-fold) in the tumor tissues (Figure 2C). Potentially, this indicates that the modification of HS chains in these U87 xenografts can occur in a different way than in the U87 tumors grown in the untreated animals, and these changes in the sulfation of the HS chains can result in the modified functional activity of HS in U87 tumors grown in DXM-treated animals.

### 2.3. Expression of Glucocorticoid Receptor in Normal and Pathological Brain Tissues

Molecular mechanism of the down-regulation of *Ndst1*, *Ndst2* and *Sulf2* expression in experimental U87 tumors is under the question, and the first thing that comes to mind is that it can be related to the functional activity of transcription factors (TFs) involved in the regulation of these genes. Since DXM is glucocorticoid and its action is realized through the interaction with glucocorticoid receptor (GR, gene *Nr3c1*) (which is a transcription factor and can regulate the activity of many different genes), we decided to investigate this TF as the potential regulator of the transcriptional activity of these genes. An analysis of the potential ability of GR to bind to the promoter regions of HS metabolism-involved genes using was performed using the ChiP-Atlas database (chip-atlas.org). Additionally, the main GR-related TFs were also included in the analysis (Table 1).

Among the HS metabolic enzymes, GR was predicted to have a maximum ability to interact with NDST1, whereas GR-related TFs JUN, JUND, FOS and STAT3 demonstrated that in regard to both NDST1 and NDST2, supporting the potential involvement of GR pathway in the transcriptional regulation of these genes. Interestingly, a similar effect has not been shown for SULF2, although this does not actually exclude an indirect influence of GR on the expression of this gene.

**Figure 3:**
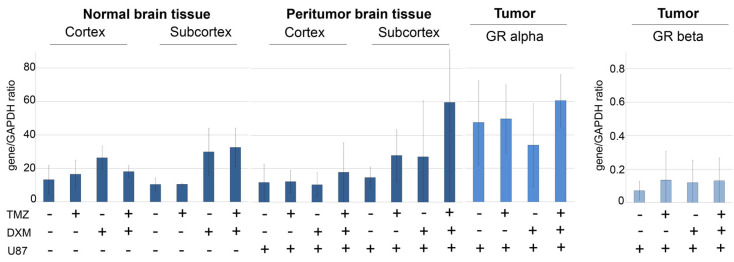
Expression of GR in normal and peritumor mouse brain tissues and experimental U87 GB tumors. For U87 xenograft tumors expression of GRalpha and GRbeta isoforms is presented. RT–PCR analysis, the intensity of the amplified DNA fragments was normalized to that of GAPDH. ANOVA + Fisher’s LSD test. TMZ—temozolomide; DXM—dexamethasone; U87—U87 human GB cells inoculated after the TMZ/DXM pre-treatment of the SCID mice.

**Figure 4:**
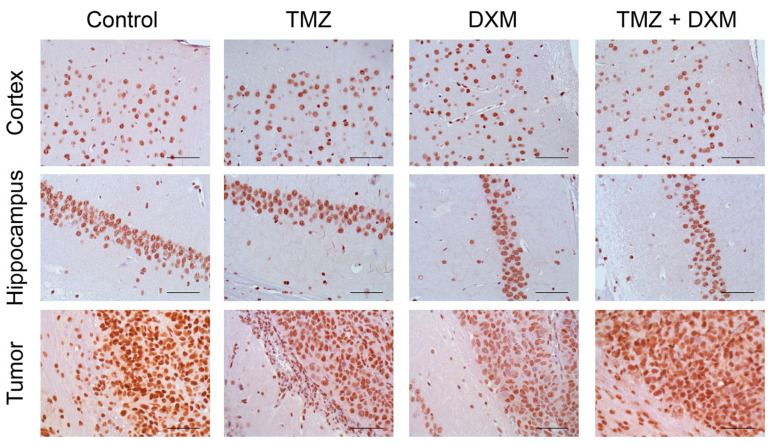
Immunohistochemical analysis of GR content in normal brain tissue and U87 GB experimental tumors. Two upper panels—mouse brain tissue (Cortex and Hippocampus)without or with TMZ/DXM. Bottom panel—U87 xenografts grown in brain of intact SCID mice and those pre-treated with TMZ and/or DXM (with adjacent mouse peritumor brain tissue). Magnification 400. Scale bars 50 µm.

For the experimental verification of this hypothesis, GR expression level was determined in normal and peritumor brain tissues as well as in the xenograft U87 tumors (Figure 3 and Figure 4), and the correlation of the GR expression levels with those for HS biosynthesis-involved genes was analyzed (Table 2).

For normal and peritumor mouse brain tissues, DXM demonstrated a tendency to up-regulate GR expression at the mRNA level up to 2–4-fold in both cortex and subcortex brain compartments, although these differences were not statistically significant (Figure 3).

The content of the GR protein molecule in the normal brain tissue showed relative stability under the pressure of TMZ and/or DXM treatments (Figure 4) supporting the RT-PCR data.

In the experimental U87 xenograft tumors, the total GR mRNA level was initially 2.5–3-fold higher than in the normal brain tissue and demonstrated a tendency to be decreased in the tumors grown in the DXM pre-treated animals, mainly due to the inhibition of expression of the GRalpha but not GRbeta isoform (Figure 3). This result is in agreement with the data of IHC staining, which shows a decrease in the content of GR protein molecules in the tumors grown in DXM pre-treated animals (Figure 4).

Although the changes in GR mRNA and protein levels were not statistically significant, we paid attention to a very large standard deviation in each experimental group and decided to study the correlation of GR expression levels with those for HS biosynthetic enzymes (Figure 2) for each individual U87 xenograft (Table 2).

Surprisingly, the xenograft U87 tumors grown in the brain of TMZ or DXM pre-treated animals demonstrated a correlation between the expression levels of GRalpha and a number of HS biosynthesis-involved genes (*Ext1/2*, *Ndst1/2*, *Glce*, *Hs2st1* and *Hs6st1/2*), unlike tumors that were grown in intact SCID mice (Table 2). Another interesting observation is that almost all statistically significant correlation coefficients were positive. At the same time, the expression level of the GR beta isoform almost did not correlate with the expression of the studied genes, and if there was such a correlation in different experimental groups, it was both the same genes (*Ext2*, *Ndst1* and *Hs2st1* for the control and TMZ + DXM groups) and other genes (*Hs6st1/2* for the TMZ group).

These results are only initial findings and raise more questions than they answer. However, they can provide the first pilot information on the existence of the glucocorticoid-GR-HS biosynthesis system-HS content/structure axis and outline promising research directions in the field of the regulation of HS biosynthesis and the coordination of the expression of HS biosynthesis-involved genes.

## 3. Discussion

According to the IHC staining of normal and peritumor mouse brain tissues, TMZ administration did not result in the changes in HS content, whereas DXM significantly reduced HS in these tissues. These results are in line with the previously shown decrease in HS content in the rat brain cortex and hippocampus [23] and, taken together, provide more strong evidence regarding the DXM effects towards HS in brain tissues.

The functional significance of such changes is still difficult to predict, but it can be assumed from the few known data on this matter. It has been shown that high HS content is associated with pro-invasive capacity of GB cells [28] and an increased HS content in GB tissues is a negative prognostic factor for GB progression [32]. From this point of view, a decrease in HS content in the normal brain tissue surrounding tumor upon DXM administration could be attributed to the positive therapeutic effect of DXM on this physiological parameter. This result complements the data of Lin [33], who showed that DXM may suppress MMP-2 secretion and cell invasion in human U87MG glioma cells and suggested that DXM can manifest its therapeutic effects not only through its influence on tumor cells but also through the modulation of the GAG composition of the brain tissue surrounding the tumor.

It is interesting that another key GAG in brain ECM chondroirin sulfate (CS) seems to have an opposite function in GB progression, which was shown in our previous work that we carried out on the same GB relapse experimental model. The treatment of organotypic rat brain slices ex vivo with TMX/DXM or chondroitinase AC resulted in the decrease in CS content and an increased adhesion, proliferation and invasion of U87 GB cells to these slices. In SCID mice, pre-treatment with TMZ/DXM led to the decrease in CS content and increased the U87 xenograft volume as well as the invasiveness of the U87 cells with the formation of extracranial tumors [25]. On the other hand, there is evidence regarding the contribution of CS to GB aggressiveness and disease progression. It can be shown that sulfated GAG antagonist surfen inhibits GB cell invasion in model system in vitro [34]. The treatment of patient-derived GB neurospheres with chondroitinase ABC in combination with TMZ in vitro resulted in a significant synergistic enhancement of GB cells killing, and the intratumoral delivery of chondroitinase ABC with TMZ enhances the survival of nude mice bearing intracranial GB30 glioma xenografts compared to each individual treatment alone [35]. These seemingly contradictory data may be caused by differences in experimental conditions—for example, the use of a non-specific blocker of all sulfated GAGS (surfen) or chondroitinase ABC instead of AC, which lead to the destruction of both CS-AC and CS-B (playing the opposite role in GB cell adhesion/invasion) [25].

The most interesting fact is that the TMZ/DXM-induced changes of HS content in brain tissue demonstrated here affect the molecular characteristics of U87 xenografts grown in brain of TMZ/DXM pre-treated animals. These delayed effects were more pronounced with the DXM administration and consisted not only of reducing the HS content but also of suppressing its biosynthetic system, mainly due to the down-regulation of N-deacetylase/N-sulfotransferases (*Ndst1* and *Ndst2*) and sulfatase 2 (*Sulf2*) expression. Unfortunately, it is not possible to compare this result with the literature data due to the lack of publications on the DXM effects on the HS biosynthesis. Indirect analogies can be made with only a few data, for example, the expression of EXT2 is significantly increased in GB [36] and its decrease under the DXM administration can be interpreted as a positive effect of DXM use, as well as in the case of a decrease in the content of HS in brain tissue. In human GB tumors, the inhibition in the transcriptional activity of HS biosynthetic system was shown, although this was due to the suppression of several other enzymes—EXT1/2 and HS6ST1/2. [37].

Molecular mechanisms behind this decrease in HS content and the down-regulation of HS biosynthetic machinery in the experimental GB tumors are unknown, but we hypothesized that the deregulation of the expression of HS biosynthesis-involved genes upon DXM administration might occur through GR. Surprisingly, although DXM-induced changes in GR expression in TMZ/DXM pre-treated mice were not statistically significant, there was a correlation of the GRalpha expression with that of a number of HS biosynthesis-involved genes (*Ext1/2*, *Ndst1/2*, *Glce*, *Hs2st1* and *Hs6st1/2*), unlike tumors that were grown in intact SCID mice. The significance of this result is not yet clear, but it highlights the need to continue working in this direction.

Another interesting observation is that the deviation in GR expression levels in normal brain tissue is relatively low, suggesting a certain control of this parameter via homeostasis mechanisms. The presence of the xenograft U87 tumor in the brain results in the evident increase in deviation for the GR expression in peritumor brain tissue, which is further intensified in U87 xenografts. This observation may indicate that during the U87 xenograft tumor development, the physiological processes of glucocorticoid-GR interplay in brain tissue can be deregulated.

## 4. Materials and Methods

### 4.1. Animals

For in vivo studies, male SCID mice (n = 64) aged 10 weeks and weighing 23–30 g were used. Animals were housed in groups of 2–5 mice in individually ventilated polycarbonate cages OptiMice (Animal Care Systems, Centennial, CO, USA) in special clean rooms with HEPA13-filtered incoming air, with free access to food and water, a 12/12 h light/dark cycle, air temperature of 22 ± 2 °C and relative humidity of 45 ± 10%. All in vivo experiments were conducted at SPF Animal Facility at the Institute of Cytology and Genetics SB RAS (Novosibirsk, Russia). All procedures were conducted in accordance with European Communities Council Directive 2010/63/EU and were approved by the Animal Care and Use Committees of the Institute of Cytology and Genetics SB RAS and FRC FTM. All efforts were made to minimize animal suffering and to reduce the number of animals used.

### 4.2. Drug Administration to Healthy SCID Mice In Vivo

The scheme of the experiment and its detailed description were presented in [25]. Briefly, 64 healthy male SCID mice were randomly divided into 4 experimental groups and treated with TMZ (MSD, Espoo, Finland) (n = 16) (intragastric administration), DXM (KRKA, Novo Mesto, Slovenia) (n = 16) (intraperitoneal injection) or both TMZ and DXM (n = 16); the control group received water intragastrically in the same volume as the TMZ group. The drugs were administered according to the following protocol: three cycles of 5 consecutive days of administration with a 9-day break between cycles (a total of 15 drug injections). The animals were weighed once a week. On the 39th day of the experiment, 6 animals from each group were sacrificed via cervical dislocation; the brains were removed, one hemisphere was divided into cerebral cortex and subcortex and collected in RNA*Later* solution (Invitrogen, Waltham, MA, USA) for RT-PCR analysis, and the second hemisphere was fixed in 10% neutral buffered formalin for 24 h at room temperature and used to prepare paraffin blocks.

The remaining pre-treated animals received an orthotopic injection of U87 human GB cells via a stereotactic inoculation of the cells into the subcortical brain structures.

### 4.3. Orthotopic U87 Experimental Tumors Development in the Pre-Treated SCID Mouse Brains

Mice were anesthetized with 1.5% isoflurane and then transferred onto a 37 °C heated operating table and placed under an anesthesia mask with 1.5% isoflurane. A 3–4 mm incision on the head skin was made in the caudal–cranial direction in the bregma area, and 5 μL of U87 cell suspension in serum-free DMEM/F12 medium (5*10^5^ cells per animal) was injected into the subcortical brain structures with a Hamilton syringe through a hole in the skull. The experimental tumors growth was monitored via magnetic resonance imaging (MRI) every 5 days, starting at day 10 after tumor cell inoculation using a BioSpec 117/16 USR horizontal tomograph (Bruker, Mannheim, Germany) at 11.7 T using a TurboRARE (Rapid Imaging with Refocused Echoes) T2 scanning sequence (TR = 2500 ms, TE_eff_ = 24 ms, NA = 5, Rare factor = 8, matrix 256 × 256 dots, field of view 2.0 × 2.0 cm). Tumor size was calculated using the Paravision 5.1 (Bruker, Mannheim, Germany) and ImageJ 1.52 software and was expressed in μL. Mice were sacrificed upon 20% weight loss; their brains were removed, and one hemisphere was divided into the cerebral cortex, subcortex and tumor and placed into RNA*Later* for RT-PCR analysis, while the other hemisphere was fixed in 10% neutral buffered formalin and used to prepare paraffin blocks.

### 4.4. Cells

The human GB U87 cell line was obtained from the Karolinska Institute (Stockholm, Sweden). The U87-RFP cell line stably expressing RFP was purchased from AntiCancer Inc., San Diego, CA, USA. Cells were maintained in IMDM medium supplemented with 2 mm L-glutamine, 100 units/mL penicillin, 100 μg/mL streptomycin and 10% fetal bovine serum at 37 °C in a humidified 5% CO_2_ incubator. For analysis, cells were harvested using trypsin/EDTA.

### 4.5. RT-PCR Analysis

Total RNA was extracted from the brain and tumor samples using the TRIzol Reagent (Thermo Fisher Scientific, Waltham, MA, USA) and RNeasy Plus Mini kit (Qiagen, Germantown, MD, USA), according to the manufacturer’s instructions. cDNA was synthesized from 1 μg of total RNA using a First Strand cDNA Synthesis kit (Fermentas, Waltham, MA, USA). Quantitative real-time RT-PCR was performed using the CFX96 Real-Time PCR Detection System (Bio-Rad, USA) and the PCR iTaq Universal SYBR Green Supermix (Bio-Rad, Hercules, CA, USA) under the following conditions: 95 °C for 2 min, followed by 40 cycles at 95 °C for 10 s and 60 °C for 30 s. The total reaction volume was 25 µL. The relative amount of mRNA was normalized against Gapdh mRNA, and the fold change for each mRNA was calculated using the 2^−ΔCt^ method. Primer sequences for human and mice genes are presented in Table 3.

### 4.6. Immunostaining

For immunohistochemistry, 3-μm sections of formalin-fixed, paraffin-embedded tissue samples were used. Deparaffinisation and antigen retrieval were performed in a PT Module with Dewax and HIER Buffer L (Thermo Scientific, Waltham, MA, USA). Tissue sections were stained using Lab Vision™ Autostainer 720-2D according to the UltraVisionQuanto HRP DAB Protocol (Thermo Scientific, Waltham, MA, USA). Briefly, sections were incubated with UltraVision Hydrogen Peroxide Block buffer for 10 min RT and then UltraVision Protein Block solution for 5 min RT and incubated with mouse anti-heparan sulfate (1:500, Millipore, Burlington, MA, USA) or anti-GR (1:5000, Abcam, Cambridge, UK) primary antibody for 1 h RT. The specificity of the stainings was verified with positive and negative controls for each primary antibody to avoid positive or false negative reactions. The signal was visualized through incubations with Primary Antibody Amplifier Quanto (10 min, RT), HRP Polymer Quanto (10 min, RT) and DAB Quanto solutions (5 min, RT). All washing steps were performed with Tris-Buffered Saline and Tween 20 buffer (Thermo Scientific, Waltham, MA, USA). Staining patterns were counterstained with hematoxylin and photographed via light microscopy with a magnification of ×400 (AxioScope.A1 with AxioCamMRc5 (Carl Zeiss, Oberkochen, Germany).

### 4.7. Dot Blots for Heparan Sulfate Content

Brain tissue samples were lysed with RIPA-buffer (Thermo Scientific, Waltham, MA, USA), containing “Complete” Protease Inhibitor Cocktail (Roche, Indianapolis, IN, USA), and was sonicated and centrifuged for 15 min at 14,000× *g*. The protein concentration was quantified using the Pierce™ BCA Protein Assay Kit (Thermo Scientific, Waltham, MA, USA). An amount of 1μg of total proteins were dot-blotted onto PVDF membranes at a volume of 1 μL. The membranes were blocked with 5% non-fat milk for 1 h and incubated with mouse anti-heparan sulfate primary antibody (1:500 Millipore, Burlington, MA, USA) overnight at 4 °C, followed by secondary peroxidase-conjugated antibodies goat anti-Mouse IgG (Abcam, Cambridge, UK) for 1 h at RT. GAGs were detected using an Optiblot ECL Detection Kit (Abcam, Cambridge, UK), according to the manufacturer’s instructions. Blots were imaged using ChemiDoc (BioRad, Hercules, CA, USA) and analyzed semi-quantitatively using ImageJ 1.52 software.

### 4.8. Statistical Analysis

ANOVA analysis with Fisher’s least significant difference (LSD) post hoc test was performed to determine statistical significance between the studied groups. The value of *p* < 0.05 was considered to indicate a statistically significant difference. Data are expressed as means ± SD. Pearson’s correlation coefficient was determined to analyze the correlation between the studied genes. All statistical analyses were performed using OriginPro 8.5 software.

## 5. Conclusions

In the GB relapse animal model, DXM administration decreases the HS content in the normal and peritumor mouse brain tissues. The xenograft GB tumors grown in the compromised microenvironment are characterized not only by a reduced HS content but also by the selective suppression of some of the HS biosynthetic enzymes, which may also reflect changes in the structure of carbohydrate HS molecules in the GB xenografts. During anti-GB radiochemotherapy, such DXM-induced decrease in HS content might contribute to the changes in the structure of the brain ECM and its interaction with residual post-surgery GB cells. 

The obtained data suggest the existence of a novel molecular mechanism through which DXM facilitates the survival of GB cells during anti-GB therapy and GB relapse development.

## Figures and Tables

**Table 1:** Predicted binding of human NR3C1 to HS metabolism-involved genes.

Target_Genes	NR3C1	JUN	JUND	STAT3	MYC	FOS	NFKB1	STAT5A
EXT1	17.50	390.33	158.21	11.84	24.97	22.73		
EXT2	6.10	109.27	25.24	38.69	19.71	73.79		6.47
NDST1	208.27	471.69	238.12	110.54	19.18	235.02	9.17	
NDST2	18.59	178.76	298.85	178.07	38.73	5.00		8.40
GLCE	19.89	61.11	41.03	43.01	25.56	47.96	23.92	
HS2ST1	6.83	67.76	103.24	16.76	48.18	45.48		17.13
HS3ST1	4.88	139.53	10.09	10.02	18.38	1.73	28.67	
HS3ST2			8.71		3.02			
HS6ST1	8.35			0.80	9.99			
HS6ST2		4.71		1.22				
HS6ST3		26.59		11.98	7.81			
SULF1	0.50	12.80	3.79	192.64	6.48	2.92		
SULF2	10.92	24.71	2.38	5.19	16.02			
HPSE	17.76	58.92	4.76	13.06	76.17			

ChiP-Atlas database; values = binding scores of MACS2 and STRING (0—white; 500—green; 1000—red, maximum).

**Table 2:** Correlation analysis of the expression of GR and HS biosynthesis-involved genes in U87 xenografts.

		GRalpha				GRbeta		
Gene	Control	TMZ	DXM	TMZ+DXM	Control	TMZ	DXM	TMZ+DXM
*biosynthesis*								
*Ext1*	0.75	0.11	0.96 *	−0.4	−0.25	0.32	−0.13	−0.09
*Ext2*	0.13	0.71	0.96 *	0.4	0.93	0.66	−0.15	0.97 *
*post-synthetic modification*								
*Ndst1*	−0.56	0.89 *	0.78	0.67	−0.54	0.15	−0.19	0.97 *
*Ndst2*	0.63	0.91 *	0.93 *	0.97 *	−0.79	0.11	−0.09	0.74
*Glce*	0.44	0.96 *	0.95 *	0.09	0.5	0.04	−0.15	0.81
*Hs2st1*	0.18	0.93 *	−0.57	0.57	−1.00 *	−0.01	−0.32	−0.35
*Hs3st1*	−0.13	0.38	−0.74	0.4	−0.89	0.96 *	0	−0.53
*Hs3st2*	−0.25	0.52	−0.73	0.41	−0.87	0.94 *	−0.08	−0.52
*Hs6st1*	0.98 *	0.75	0.63	0.79	−0.35	−0.31	0.61	0.92
*Hs6st2*	0.93	0.92 *	0,76	−0.39	0.09	−0.12	−0.18	0.49
*degradation*								
*Sulf1*	−0.3	0.74	−0.78	0.68	−0.85	−0.44	0.03	−0.22
*Sulf2*	0.63	0.77	0.85	0.87	0.63	−0.33	−0.19	0.9
*Hpse*	0.62	0.77	0	0.88	0.58	0.55	0.53	0.86

* Pearson’s correlation coefficient was calculated pairwise for GRalpha and GRbeta isoforms and HS biosynthetic genes, statistically significant results (*p* < 0.05) are marked with an asterisk.

**Table 3:** Sequences of primers used in PCR analysis.

Gene	Organism	Sequences
*EXT1/Ext1*	Human	F 5′-TTGGGTCCTTCAGATTCCTG-3′ R 5′-TCCTCCAGGATGTTTGTTCC-3′
	Mus musculus	F 5′-AGCACAAGGATTCTCGCTGT-3′ R5′-GGAACCAGACAGAAAGTGGC-3′
*EXT2/Ext2*	Human	F 5′-AAGCACCAGGTCTTCGATTACC-3′ R 5′-GAAGTACGCTTCCCAGAACCA-3′
	Mus musculus	F5′-ACATCCCACAGAGGCAGATT3′ R5′-GATCTGTAGGGTGGCCAGAG3′
*NDST1/Ndst1*	Human	F 5′-CACACAGAACGAACTACGC-3′ R 5′-CCCGTTGATGATCTTGTCC-3′
	Mus musculus	F5′-TATGTCAACCTGGATGCCTG-3′ R5′-CACTCAGCAGGCTGTTCTCA-3′
*NDST2/Ndst2*	Human	F 5′-GCCTCCAGTTCCACCTC -3′ R 5′-CGACGAAGAACTGGTCC-3′
	Mus musculus	F5′-TGGTCCAAGGAGAAAACCTG-3′ R5′-GCAGGCTCAGGAAGAAGTGA-3′
*GLCE/Glce*	Human	F5′-CTACACAATGGGGACCTCAAGGC-3′ R5′-GCCACCTTTCTCATCCTGGTTCC-3′
	Mus musculus	F5′-GCTCGCTTCAGTTTTCCTCA-3′ R5′-TCTTAGTACATTTCTGGCTTCAATTC-3′
*HS2ST1/Hs2st1*	Human	F5′-CCAGATCCAGAAACTGGAGG-3′ R5′-TCCATTGTATGTCGCTGCTC-3′
	Mus musculus	F5′-TCTTGGAGAACCAGATCCAGA3′ R5′-ATGGCGCTGTTCAATTTCTC3′
*HS3ST1/Hs3st1*	Human	F5′-CGGGTCTCAGTGGGTGCCTG-3′ R5′-ATCCTGGAGGGTCCCCGCTT-3′
	Mus musculus	F5′-GGAGGAGCATTACAGCCAAG-3′ R5′-TTTGGGCGAAGTGAAATAGG-3′
*HS3ST2/Hs3st2*	Human	F5′-ACCCCACTTCTTTGACAGGA-3′ R5′-CAAAGTAGCTGGGCGTCTTC-3′
	Mus musculus	F5′-AACTACGGACGAGGACTGGA-3′ R5′-ATTACCTCTGGGGCAAATCC-3′
*HS6ST1/Hs6st1*	Human	F5′-CGACTGGACCGAGCTCAC-3′ R5′-GGTCTCGTAGCAGGGTGATG-3′
	Mus musculus	F5′-TGGCTCTTCTCTCGCTTCTC-3′ R5′-GTCTAGCACACCGGGCAC-3′
*HS6ST2/Hs6st2*	Human	F5′-TCACCAGCTGTGTGCCC-3′ R5′-GTGTCGGAGGATGGTGATGT-3′
	Mus musculus	F5′-CCAGGCTGAGACCTTCCAG-3′ R5′-TGTGGAGGATGGAGAGTTGG-3′
*SULF1/Sulf1*	Human	F5′-CTCACAGTCCGGCAGAGCAC-3′ R5′-CACGGCGTTGCTGCTATCTGC-3′
	Mus musculus	F5′-CCTTGCAGGGAAGCTTCAAA-3′ R5′-GCTGAGTTCTGGGAGCTTGA-3′
*SULF2/Sulf2*	Human	F5′-GAGGCAGATTCACGTCGTTTCCA-3′ R5′-ATCTGGTGCTTCTTTTGGGATGCGGGAG-3′
	Mus musculus	F5′-GTTCCTCCCGCGATCTAGC-3′ R5′-GTGTCGTGAGGATGGGATTC-3′
*HPSE/Hpse*	Human	F5′-TTCGATCCCAAGAAGGAATC-3′ R5′-ATAAAGCCAGCTGCAAAGGT-3′
	Mus musculus	F5′-GGCTAGAGGCTTATCTCCTGC-3′ R5′-TCTTTCTTCGGAAGTCGGTT-3′
*GAPDH/Gapdh*	Human	F5′-GGGCGCCTGGTCACCAG-3′ R5′-AACATGGGGGCATCAGCAGAG-3′
	Mus musculus	F5′-CGTCCCGTAGACAAAATGGT-3′ R5′-TTGATGGCAACAATCTCCAC-3′

## Data Availability

The original contributions presented in the study are included in the article. Further inquiries can be directed to the corresponding author.
